# *nBio*Chip, a Lab-on-a-Chip Platform of Mono- and Polymicrobial Biofilms for High-Throughput Downstream Applications

**DOI:** 10.1128/mSphere.00247-17

**Published:** 2017-06-28

**Authors:** Anand Srinivasan, Nelson S. Torres, Kai P. Leung, Jose L. Lopez-Ribot, Anand K. Ramasubramanian

**Affiliations:** a Department of Biomedical Engineering, The University of Texas at San Antonio, San Antonio, Texas, USA; b BioBridge Global, San Antonio, Texas, USA; c Dental and Craniofacial Trauma Research and Tissue Regeneration, U.S. Army Institute of Surgical Research, Fort Sam Houston, Texas, USA; d Department of Biology, The University of Texas at San Antonio, San Antonio, Texas, USA; e Department of Biomedical, Chemical and Materials Engineering, San Jose State University, San Jose, California, USA; Carnegie Mellon University

**Keywords:** high-throughput screening, antimicrobial agents, biofilms

## Abstract

Current *in vitro* techniques for the culture of microorganisms, and particularly of delicate microbial biofilms, are still mostly limited to low-density plates and manual labor and are not amenable to automation and true high-throughput (HT) applications. We have developed a novel fully automated platform for the formation of mono- and polymicrobial biofilms of *Staphylococcus aureus*, *Pseudomonas aeruginosa*, and *Candida albicans* at the nanoscale level. The *nBio*Chip is robotically printed, robustly handled, and scanned using a standard microarray reader. Using this technique, hundreds to thousands of identical nanobiofilms encapsulated in hydrogel spots were cultured on microscope slides. The spots can withstand the washing steps involved in screening assays. The miniaturized biofilms demonstrated characteristics similar to those displayed by conventionally formed macroscopic biofilms, including (i) three-dimensional architectural features, (ii) synthesis of exopolymeric matrix material, and (iii) elevated resistance to antibiotic treatment. On the basis of our results, the *nBio*Chip can generate reliable high-throughput antimicrobial susceptibility testing (HT-AST) in 12 to 18 h. The chip serves as a proof-of-concept universal platform for high-throughput drug screening and other downstream applications and furthers understanding of microbial interactions in mixed-species communities at the nanoscale level.

**IMPORTANCE** With an estimated 80% of infections being associated with a biofilm mode of growth and the ensuing recalcitrance of these biofilms with respect to conventional antibiotic treatment leading to high mortality rates, there is a dire and unmet need for the development of novel approaches to prevent, treat, and control these infections. Both bacteria and fungi are capable of forming biofilms that are inherently fragile and often polymicrobial in nature, which further complicates treatment. In this work, we showcase a nanobiofilm chip as a convenient platform for culturing several hundreds of mono- or polymicrobial biofilms and for susceptibility testing. This platform enables true ultra-high-throughput screening for antimicrobial drug discovery or diagnostics or for addressing fundamental issues in microbiology.

## Abstract (precis)

With an estimated 80% of infections being associated with a biofilm mode of growth and the ensuing recalcitrance of these biofilms with respect to conventional antibiotic treatment leading to high mortality rates, there is a dire and unmet need for the development of novel approaches to prevent, treat, and control these infections. Both bacteria and fungi are capable of forming biofilms that are inherently fragile and often polymicrobial in nature, which further complicates treatment. In this work, we showcase a nanobiofilm chip as a convenient platform for culturing several hundreds of mono- or polymicrobial biofilms and for susceptibility testing. This platform enables true ultra-high-throughput screening for antimicrobial drug discovery or diagnostics or for addressing fundamental issues in microbiology.

## INTRODUCTION

Biofilm-associated infections (BIs) are notoriously difficult to treat as they demonstrate 100-fold to 1,000-fold increases in antimicrobial resistance compared to their planktonic counterparts (1). BIs are the main cause of morbidity and mortality associated with biomedical-device-related infections, adding over 1 billion dollars to hospitalization costs annually in the United States alone (2). Biofilms are three-dimensional (3D), dynamic microbial communities consisting of attached cells encased in a self-produced exopolymeric matrix (3). The cells within the biofilms show increased antibiotic resistance through multiple mechanisms and are protected from the host defenses due in part to the presence of extracellular matrices, thus making biofilm infections most difficult to treat (4, 5). Experimental models of biofilms that mimic the natural environment provide a convenient way to understand biofilm biology. In a natural disease setting, biofilms are formed when cells adsorb to surfaces (such as implantable catheters) that are coated with host serum proteins, replicate, and release an exopolymeric matrix that encases the cells (6, 7). Several *in vitro* experimental models simulate this *in vivo* process where the cells are attached to a two-dimensional surface precoated with plasma or a protein of interest (8). Abiotic surfaces such as flasks, well plates, or filters are coated with serum proteins to initiate cell adhesion, and the growth conditions are optimized to facilitate the process of biofilm formation (9, 10). Although these models have been useful in expanding our knowledge of biofilms, some of the major disadvantages are that these models incorporate low-throughput processes and that the inherently fragile biofilms require delicate handling during the washing and analysis steps, thus challenging the high-throughput automation, reproducibility, and reliability of the biofilm assays (11). To address these issues, we recently developed a novel platform for fungal biofilm culture consisting of *Candida albicans* cells encapsulated in nanoliter volumes of hydrogel matrices on glass slides in a microarray format (12). We demonstrated that the advantages of this high-throughput fully automated platform include (i) production of hundreds of spatially distinct but identical “nanobiofilms” on a single glass slide; (ii) formation of biofilms displaying phenotypic properties comparable to those of macroscopic biofilms; (iii) the possibility of culturing of cells for prolonged periods of time without additional media; (iv) firm attachment of biofilms to the substrate without detachment against multiple washings; and (v) rapid and sensitive fluorimetric analyses.

In this work, we expanded the use of our platform to the culture of mono- and dual-species bacterial biofilms at the nanoscale level and also of mixed bacterium-fungus biofilms. To demonstrate the versatility of our platform, we cultured both Gram-positive (*Staphylococcus aureus*) and Gram-negative (*Pseudomonas aeruginosa*) bacteria. *S. aureus* is the leading cause of nosocomial infections, since, as a commensal, *S. aureus* can easily colonize indwelling catheters and biomedical devices and can have easy access to systemic circulation and the vitals (13). *P. aeruginosa* biofilms cause pulmonary infections in cystic fibrosis patients (14–16). Infections due to polymicrobial biofilms have also been found to correspond to significantly higher mortality rates (70%) than are seen with infections caused by a single species of microorganism (23%) (17, 18). Among the nosocomial infections that are polymicrobial in nature, *S. aureus*, *C. albicans*, and *P. aeruginosa* were identified as the most commonly occurring microorganisms contributing to the high morbidity and mortality rates associated with such infections (19, 20). Hence, this nanobiofilm platform provides versatility and flexibility suitable for the formation of bacterial and fungal as well as polymicrobial biofilms and allows the implementation of ultra-high-throughput applications, including susceptibility testing and screening for novel antibiotics, which might otherwise be impossible to achieve using traditional culture systems.

## RESULTS

For any given microorganism, the successful fabrication of a nanobiofilm microarray requires a clear definition of the specifications of the needs of the platform and the proper design to meet those specifications. Briefly, the key specifications are that the chip should hold firmly several hundreds of spatially distinct and robust biofilms resembling conventional macroscale biofilm cultures and should enable rapid, reliable, and reproducible analyses of these biofilms with a standard microarray scanner. These specifications were achieved using a factorial design of experiments wherein the appropriate combinations of abiotic and biotic variables were determined for optimal biofilm culture and analysis, as described before by our group (21). These principles guided the development of the bacterial biofilm chips described below.

### *S. aureus* nanobiofilm chip.

Biofilm formation depends on several factors such as the composition, pH, ionic strength, and temperature of media and the physicochemical properties of the substrate (9, 22, 23). In case of biofilm microarrays, the 2D substrate is replaced by the 3D encapsulating hydrogel. To obtain fully formed biofilms within self-supporting hemispherical hydrogel spots, we optimized the culture conditions by employing a two-level factorial design method described in detail elsewhere (21). The variables that were optimized include the growth conditions (pH and temperature), hydrogel matrix (type, strength, and concentration), media (type, concentration, and combination), and the seeding cell concentrations appropriate for the maximal biofilm yield necessary to generate reproducible results. To ascertain cell growth and biofilm formation, the *S. aureus* nanobiofilms were stained for cell viability and extracellular matrix and were visualized by confocal microscopy.

**FIG 1:**
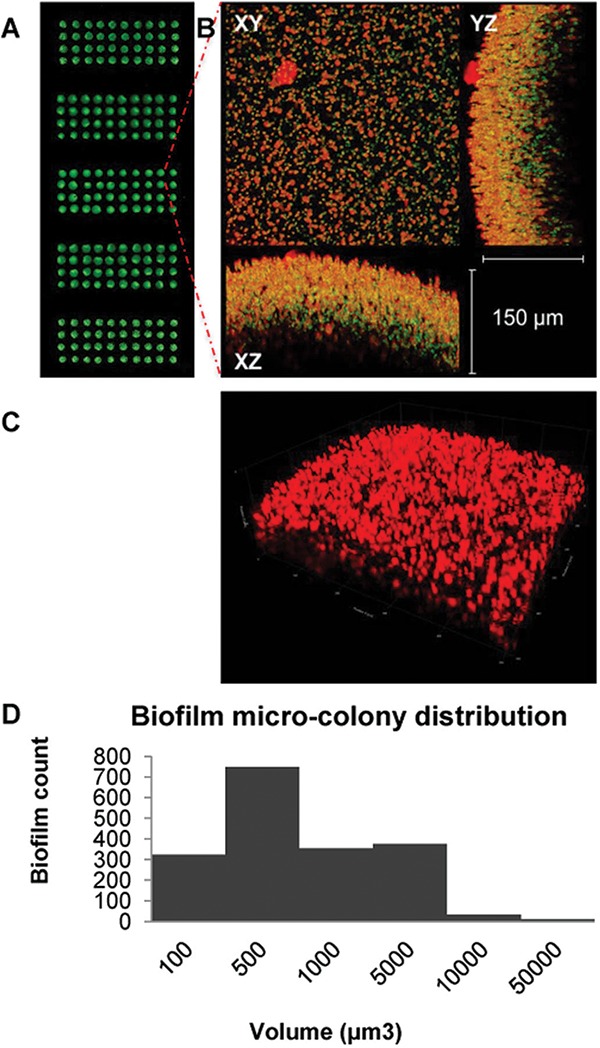
A biofilm chip of an *S. aureus* nanoscale biofilm. (A) Microarray scanner image of *nBio*Chip with identical biofilms of *S. aureus*. The biofilm spots are interspaced at 1 mm with a diameter of 500 μm. (B) Fluorescence micrographs of a single spot of *S. aureus* nanobiofilms stained with SYTO-9 and SYPRO Ruby in the *xy*, *yz*, and *xz* axes. SYTO-9 labels the cell in green, and SYPRO Ruby binds to the exopolymeric matrix material, appearing in red. The spot appears to contain numerous microcolonies of *S. aureus* biofilms. The thickness of the biofilm calculated for the *yz* and *xz* axes is 150 μm. (C) A confocal laser scanning micrograph of a biofilm spot stained with SYPRO Ruby, demonstrating the exopolymeric matrix material produced by the biofilm. (D) Frequency distribution of the microcolonies of various diameters within a single nanobiofilm spot.

Under these conditions, we formed a nanobiofilm microarray of *S. aureus* containing up to 1,200 spots, each 30 nl in volume. Cell viability and distribution uniformity after 24 h of culture were established using fluorescent staining with a metabolic dye, which also binds to bacterial cells (24) (Fig. 1). We also extensively characterized the resulting *S. aureus* nanobiofilms by confocal microscopy. We observed that the cells were distributed homogeneously at each spot and that they produced abundant exopolymeric matrix, as seen by the green fluorescence of SYTO-9 from viable cells and red fluorescence of SYPRO Ruby from exopolymeric matrix. The biofilms also had a characteristic 3D architecture, reaching a thickness of about 150 μm.

The *S. aureus* cells within each nanobiofilm culture formed characteristic 3D spherical microcolonies that were densely packed at approximately 2,000 colonies per μm^3^ volume of the gel. Since the cells were seeded at a density of 500 cells per spot with a volume of approximately 1 μm^3^, the biofilm culture data determined in the absence of any addition of external media confirmed that the culture conditions were favorable for the growth of *S. aureus* for a period of 24 h. More than 90% of the microcolonies grew isometrically during the incubation time and varied in size, with a median volume of 500 μm^3^ or a diameter of around 10 μm, indicating that each of these microcolonies might have consisted of a few hundred cells. Some microcolonies were as large as 10,000 μm^3^, which might have represented several thousand cells or, alternatively, a few colonies that had coalesced together during growth. Since the exopolymeric matrices are secreted by and associated with the individual cells, as expected, the distribution of exopolymeric matrix in the biofilm spot followed a trend similar to that seen with the microcolonies. We also observed that the exopolymeric materials (EPM) were ~2-fold larger than the microcolonies, as measured by the ratio of the fluorescence intensity of SYPRO Ruby to that of SYTO-9, suggesting that the cells secrete abundant amounts of matrix components which may also help in communication within the microbial community.

### Susceptibility of *S. aureus* nanobiofilms to antibiotics.

**FIG 2:**
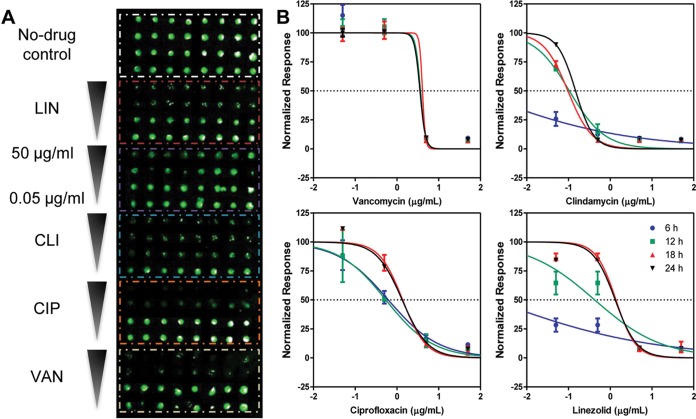
Antimicrobial susceptibility testing (AST) for inhibition of *S. aureus* biofilm formation. (A) Microarray scanner image of *S. aureus nBio*Chip after antimicrobial drug treatment with linezolid (LIN), clindamycin (CLI), ciprofloxacin (CIP), and vancomycin (VAN). The antimicrobial drugs are arrayed at decreasing concentrations of 50, 5, 0.5, and 0.05 μg/ml, with multiple replicates for each condition. (B) Antimicrobial profile of susceptibility of *S. aureus* nanobiofilms to antibiotics preventing biofilm formation. The data represent dose-response profiles of *S. aureus* with respect to VAN, CLI, CIP, and LIN at 6, 12, 18, and 24 h.

In contrast to the problems commonly found with cell detachment in plate-based biofilm assays (25, 26), encapsulation makes the nanobiofilm spots resistant to multiple washings, and the biofilm chip is particularly useful to test the effect of drugs on these biofilms. Further, the chip provides a high-throughput platform to test multiple drugs and doses simultaneously (12). Thus, we next tested several antibiotics for their ability to inhibit the formation of *S. aureus* nanobiofilms, including four drugs representing four different classes of antibacterial antibiotics: clindamycin (macrolide), ciprofloxacin (fluoroquinolone), linezolid (oxazolidinone), and vancomycin (glycopeptide). We tested the susceptibility of nanobiofilms for various incubation periods between 6 and 24 h in order to ascertain if reduction to nanoliter volumes may be particularly advantageous in shortening the duration of the antibacterial susceptibility assays, a highly desirable characteristic that should decrease the time required to obtain test results. Interestingly, we observed that the susceptibility profiles at 6, 12, 18, or 24 h after exposure were substantially different for the four drugs (Fig. 2). The biofilms showed a more gradual dose-response curve with ciprofloxacin, clindamycin, and linezolid but a much steeper response curve with vancomycin. The all-or-none response of the biofilms to vancomycin confirms the high potency of vancomycin against *S. aureus* and other Gram-positive bacteria due to its inhibitory effect on cell wall biosynthesis. In addition, Fig. 2 also indicates that, while vancomycin and ciprofloxacin are effective within 6 h of exposure, the biofilms need to be exposed to clindamycin and linezolid for at least 12 h before they demonstrate efficacy. Both clindamycin and linezolid are bacteriostatic drugs, which prevent cell growth by inhibiting protein synthesis by binding to the 30S/50S subunit of the ribosome (27), and consequently need more time to manifest any discernible effects of the biofilms. However, irrespective of the bactericidal or bacteriostatic action of the drugs, Fig. 2 shows that 12 to 15 h is sufficient to obtain an MIC value comparable to that seen with the standard 24-h drug exposure in a 96-well plate. Of note, such a substantial reduction in assay duration not only may accelerate the throughput in drug discovery programs but also may be particularly crucial in antibiotic susceptibility testing (AST), where rapid and reliable prediction can decide the clinical outcome (28).

### *P. aeruginosa* nanobiofilm chip.

**FIG 3:**
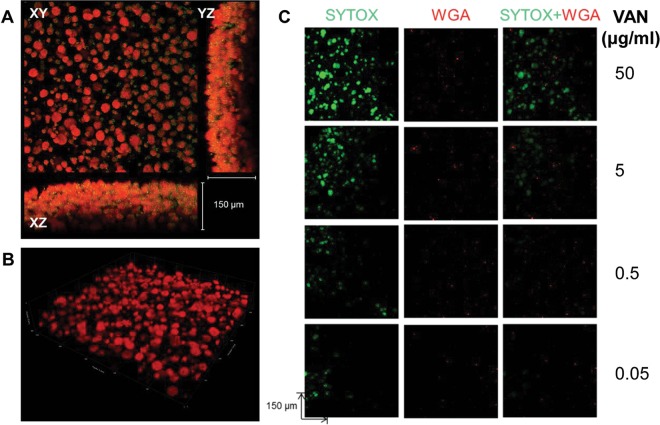
Nanoscale biofilm of *P. aeruginosa*. (A) Fluorescence micrograph of a single spot of *P. aeruginosa* nanobiofilm stained with SYTO-9 and SYPRO Ruby. SYTO-9 labels the cell in green, and SYPRO Ruby binds to the exopolymeric matrix material, appearing in red. The spot appears to contain microcolonies of *P. aeruginosa* biofilms suspended in the hydrogel encapsulating the biofilm. The thickness of the biofilm calculated in the *yz* and *xz* axes is 150 μm. (B) A confocal laser scanning micrograph of a biofilm spot stained with SYPRO Ruby, demonstrating abundant exopolymeric matrix material produced by the biofilm. (C) Fluorescent micrographs demonstrating AST of *P. aeruginosa* biofilms stained with SYTOX and wheat germ agglutinin (WGA). SYTOX labels the dead cells in green, and WGA represents a control dye (red). The micrographs represent the profile of the response of *P. aeruginosa* to VAN at 50, 5, 0.5, and 0.05 μg/ml.

Next, we developed a bacterial biofilm chip for use with a common Gram-negative opportunistic pathogen, *Pseudomonas aeruginosa*. Initial parameters were optimized using factorial design as described before. In its final format, the *P. aeruginosa* nanobiofilm chip consisted of up to 1,200 spots of 30 nl each on a glass slide. We observed that fully grown *P. aeruginosa* biofilms had a 3D architecture consisting of multiple microcolonies (Fig. 3) comparable to those cultured in conventional *in vitro* models (29, 30). The cells were fairly dense, with 200 colonies per mm^3^, and the median volume and diameter of these microcolonies were ~5,000 μm^3^ and 20 μm, respectively. Again, the exopolymeric matrix distribution, as visualized by SYPRO Ruby, followed a trend similar to that seen with the microcolony distribution, suggesting that the former was closely associated with the latter (see Fig. 5). Thus, in contrast to the *S. aureus* biofilms, the individual colonies in *P. aeruginosa* biofilms were larger and less densely packed. Once conditions for the formation of *P. aeruginosa* biofilms were fully established, we performed susceptibility testing to examine the ability of different antibiotics—ciprofloxacin, clindamycin, linezolid, and vancomycin—to inhibit the formation of these nanobiofilms (data not shown). As expected, vancomycin was not effective against Gram-negative *P. aeruginosa* (31). Also consistent with well plate assays, the bacteriostatic drugs clindamycin and linezolid were also ineffective against the formation of *P. aeruginosa* biofilms (32, 33). Ciprofloxacin was the only drug that was effective in inhibiting *P. aeruginosa* biofilm formation (50% inhibitory concentration [IC_50_] = 0.062 μg/ml ± 0.98 μg/ml). The utility of FUN-1 staining as a measure of metabolic activity for susceptibility testing using the microarray scanner was further benchmarked with the fluorescence micrographs of the chip treated with vancomycin and stained using SYTOX and wheat germ agglutinin (WGA) (Fig. 3C).

### *S. aureus*-*P. aeruginosa* polymicrobial nanobiofilm chip.

**FIG 4:**
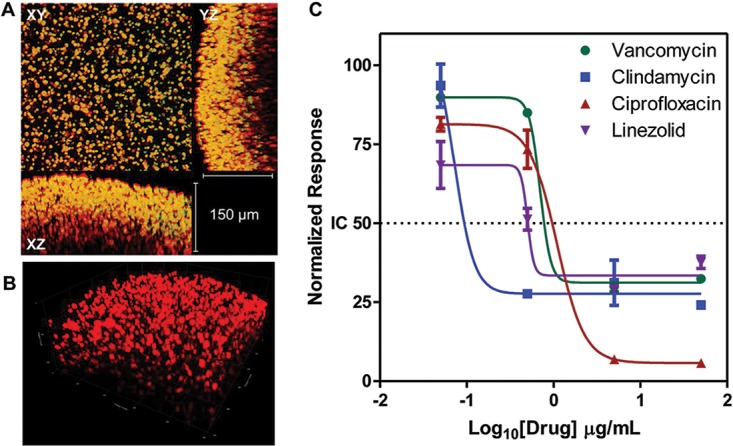
Nanoscale mixed-species biofilm of *S. aureus* and *P. aeruginosa*. (A) Fluorescence micrographs of a single spot of mixed-species nanobiofilms stained with SYTO-9 and SYPRO Ruby. The thickness of the biofilm calculated in the *yz* and *xz* axes is 150 μm. SYTO-9 labels the cells of *S. aureus* and *P. aeruginosa* in green, and SYPRO Ruby binds to the exopolymeric matrix material, appearing in red. (B) A confocal laser scanning micrograph of a biofilm spot stained with SYPRO Ruby, demonstrating exopolymeric matrix material produced by the biofilm. (C) Profile of susceptibility of mixed-species biofilms of *S. aureus* and *P. aeruginosa* to antimicrobial agents in preventing biofilm formation. The data represent profiles of susceptibility of monospecies *P. aeruginosa* biofilms and mixed-species biofilms to VAN, CLI, CIP, and LIN at 24 h.

*S. aureus* and *P. aeruginosa* are commonly found together in many wound infections, and understanding the dynamics of the microbial interactions is important for effective treatment (18, 20). Hence, we designed a polymicrobial biofilm chip consisting of a mixed culture of these two organisms. Starting with equal numbers of *S. aureus* and *P. aeruginosa* in seed cultures, we analyzed biofilm formation after 24 h with confocal microscopy. We stained *S. aureus* and *P. aeruginosa* using FUN-1 and Sypro Ruby to detect the microbial population and exopolymeric matrix content in the mixed biofilm, respectively. As shown in Fig. 4, the mixed cultures formed thick 3D biofilms with segregated microcolonies of both organisms with copious extracellular matrix production. Interestingly, all four drugs were effective in mixed biofilm cultures, with dose-response curves comparable to those seen with *S. aureus* biofilms. We believe that this effect was due to the static environment in the hydrogel compared to the free-floating environment in a well plate or a test tube containing liquid media. One such observation of increased susceptibility to ciprofloxacin was reported in the case of *Staphylococcus-Pseudomonas* coculture infection in an *in vivo* wound model (34).

**FIG 5:**
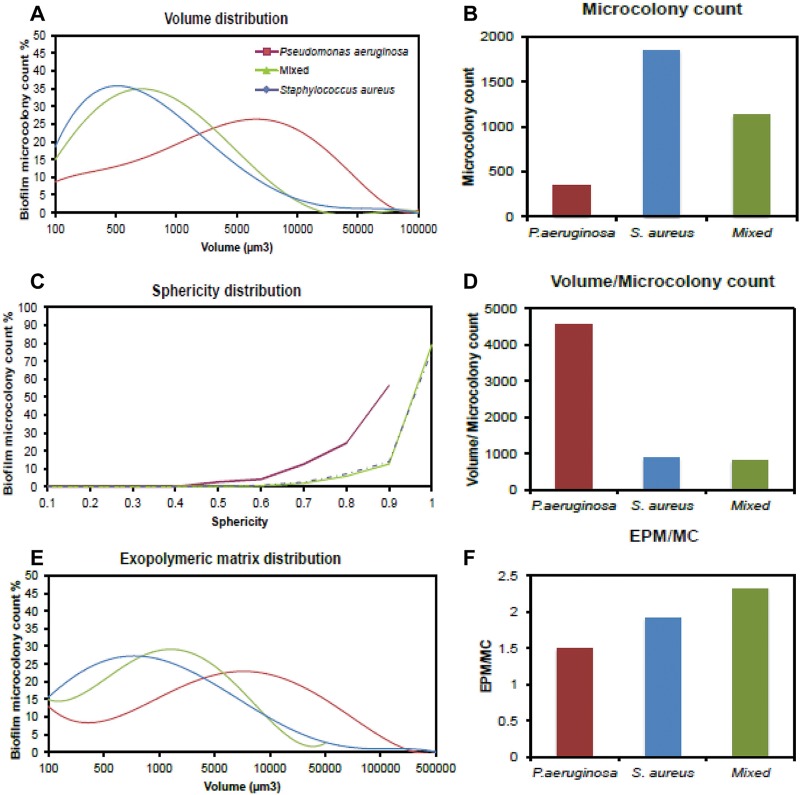
Characterization of mono- and mixed-species biofilms of *S. aureus* and *P. aeruginosa* on the chip. (A) The microcolonies of mono- and mixed-species biofilms ranged in size from 100 to 100,000 μm^3^. (B) The numbers of colonies formed are intrinsic to the type of microorganism and association. *S. aureus* produced the highest number of microcolonies. (C) The microcolonies of *S. aureus* and mixed-species biofilms are mostly spherical in shape, and *P. aeruginosa* formed disc-shaped (ellipsoid) microcolonies. (D) The volumetric size of the microcoloines is antithetical to colony counting. *P. aeruginosa* demonstrated the highest volume/microcolony count ratio. (E) A profile of the exopolymeric matrix material produced by mono- and mixed-species biofilms. (F) Mixed-species biofilms manifested the highest level of exopolymeric matrix material (EPM) production relative to the microcolony count (MC).

In addition to antimicrobial susceptibility testing, the *nBio*Chip facilitates analyses of characteristics of mixed-species biofilms such as the number, volume, and geometric distribution of microcolonies, as well as the extent of exopolymeric matrix production (Fig. 5). Investigation of compactness and spatial and population heterogeneity within a polymicrobial biofilm provides fundamental understanding of multispecies interactions. As seen in Fig. 5A to C, *S. aureus* cultures and mixed cultures demonstrated sphericity and high microcolony counts, with a majority of microcolonies showing sizes of under 500 and 1,000 μm^3^. On the other hand, *P. aeruginosa* formed more-ellipsoid colonies at a higher volume of 5,000 μm^3^. The volume of the exopolymeric matrix material calculated on the basis of the number of microcolonies per unit volume highlights the amicability of growth conditions and synergy between species in mixed cultures. As shown in Fig. 5D to F, it is obvious that *S. aureus* cultures and mixed cultures exhibited similar trends in the distribution of exopolymeric matrix production in the hydrogel spots. Nevertheless, the mixed cultures displayed a greater amount of exopolymeric matrix per total volume of microcolonies formed in the spot. Of note, the composition of exopolymeric matrix may influence the drug susceptibility of biofilms due to either physicochemical drug-matrix interactions or biological responses of the cells to the environment (35). All this information enables greater control in developing assays with suitable media, buffer, cell-seeding density, seeding ratio, choice of hydrogel, growth factors, etc., to create microenvironments that more closely mimic the clinical situation; resulting in “*in vivo*-like” assays.

### *S. aureus*-*C. albicans* polymicrobial nanobiofilm chip.

**FIG 6:**
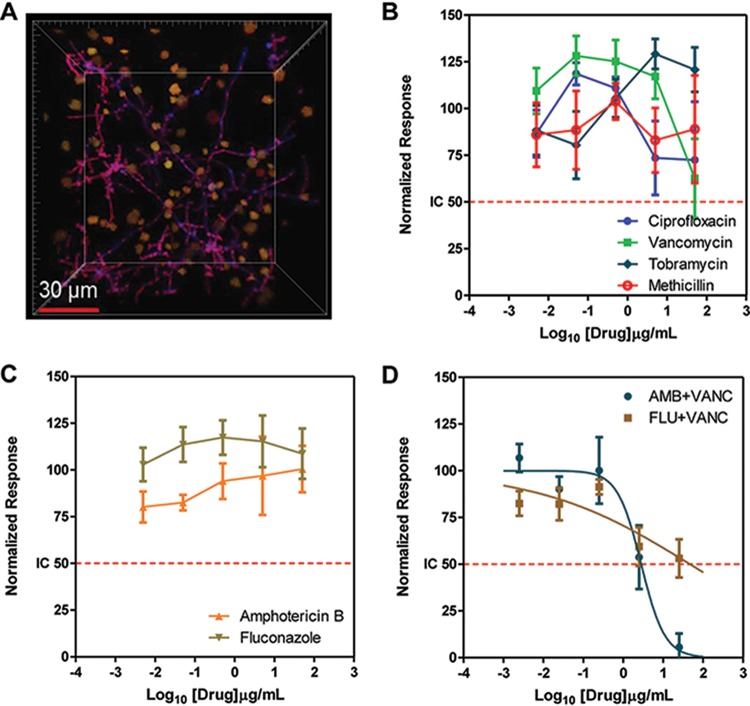
Nanoscale mixed-species biofilm of *S. aureus* and *C. albicans* on chip. (A) Fluorescence micrographs of a section of the spot containing mixed-species nanobiofilms stained with FUN-1 and concanavalin A. Staining with FUN-1 demonstrated all viable fungal and bacterial populations (in orange-yellow), and concanavalin A stained only fungal cell walls (in blue). (B to D) Profile of susceptibility of mixed-species biofilms of *S. aureus* and *C. albicans* to antibiotics (B), antifungals (C), and combination treatment (D). The data represent dose-response profiles of mixed-species biofilms with respect to ciprofloxacin, vancomycin, tobramycin, and methicillin (B) and to amphotericin B and fluconazole (C) at 50, 5, 0.5, 0.05, and 0.005 μg/ml. (D) Profile of susceptibility to combinations of 25 μg/ml of vancomycin (VANC) with 25, 2.5, 0.25, 0.025, and 0.0025 μg/ml of amphotericin B (AMB) and fluconazole (FLU).

Along with *S. aureus*, the opportunistic pathogenic fungus *C. albicans* ranks among the most frequent causative agents of nosocomial infections, responsible for high morbidity and mortality rates in hospitalized patients (36, 37). The synergistic interactions between these organisms increase the severity of disease and complicate treatment of biofilm-associated infections; hence, understanding the interactions between *S. aureus* and *C. albicans* is important to combat polymicrobial infections (36, 38). We previously developed a monospecies *C. albicans* biofilm chip and evaluated the efficacy of antifungal drugs against biofilms. In this work, we developed a dual-microbial biofilm chip for nanoscale cultures of *S. aureus* and *C. albicans*. We seeded equal numbers of *S. aureus* and *C. albicans* in media optimized for the growth of both *S. aureus* and *C. albicans*, using factorial design as described previously (22). After 24 h, the nanobiofilms were stained separately for *S. aureus* and *C. albicans* and were analyzed by confocal microscopy. As shown in Fig. 6, mixed biofilms of *C. albicans* and *S. aureus* were composed of filamentous *C. albicans* and interspersed microcolonies of *S. aureus*. Because of the encapsulation, the nanobiofilms have an architecture that is different from that of the complete covering of *C. albicans* filaments by *S. aureus* colonies, as previously reported in free-floating biofilms (39).

Next, we tested the antimicrobial susceptibility of the mixed *C. albicans*-*S. aureus* biofilms using antibacterials or antifungals or combinations by estimating total cell viability after exposure to the antibiotics. We observed that none of the four antibacterials (ciprofloxacin, methicillin, tobramycin, and vancomycin) were effective in reducing the total biofilm load since the antibacterials target only the bacteria, leaving the fungi alive. Similarly, the two antifungals (amphotericin and fluconazole) were not effective since these drugs target only *C. albicans* without affecting *S. aureus*. This response could be attributed to *C. albicans* conferring antimicrobial resistance to *S. aureus* in a mixed-species biofilm, a phenomenon also reported by Harriott and Noverr (36). We also tested a combination therapy consisting of an antifungal (amphotericin or fluconazole) with an antibiotic (vancomycin). As expected, the combination of fluconazole with vancomycin was not effective since *C. albicans* biofilms are known to be resistant to fluconazole (40). In contrast, treatment with amphotericin B and vancomycin was effective against both *C. albicans* and *S. aureus*, although we note here that the concentrations of amphotericin B that were effective are considered toxic. Our data confirm that mixed bacterium/fungus infections can be treated effectively only by targeting all the constituents of the polymicrobial biofilms.

## DISCUSSION

Techniques to culture microorganisms for *in vitro* phenotypic assays have generally lagged behind other technological advancements in the last two centuries. Although microorganisms are in the size range of few micrometers, it has become a common practice to grow them in macroscale using Erlenmeyer flasks, petri dishes, and agar plates (41). In this era of advanced automation and robotics, we still refer to the well plate as the ultimate high-throughput platform (42). Although the densities of plates have increased from 96 to 1,536 wells per plate, some assays demand 96-well or 384-well plates to produce data of sufficient quality (43). The quality of data depends on factors such as signal intensity, signal-to-noise ratio, and Z′ factor (44). The high-density plates require proportionally more concentrated biological reagents to achieve sufficient signal intensity and data quality. Especially in the case of culturing microbial biofilms, we are often restricted to the use of 96-well plates because of the intricacies of the techniques involved in the assay. Even though liquid-handling stations can aid in the initial dispensing of cells into the wells, it is almost impossible to eliminate the need for human intervention to conduct the rest of the assay. Some of the factors that burden full automation of these assays are (i) the delicate nature of biofilms, (ii) the three-dimensional architecture, (iii) diverse morphologies, (iv) potential disruption, and (v) the loss of cells during the washing steps involved in the assay.

To circumvent all these issues, our group has developed the *nBio*Chip, a fully automated platform to handle biofilm and planktonic culturing in nanoscale volumes. The chip is 1 in. by 3 in. in size and in its full potential can encompass up to 2,000 spots, where each “spot” is equivalent to a “well” in a well plate. The printing procedure involves the use of a robotic arrayer to dispense nanoliter volumes of spots containing a mixture of hydrogel, media, buffer, and cells, giving rise to identical biofilms. The biofilms grow inside the hydrogel, which is firmly attached to the chip, resulting in a robust biofilm chip. Therefore, robotic handling of any assay procedures such as liquid aspiration and dispensation, washing, and rinsing does not lead to biofilm disruption or loss of cells. The endpoint of the chip-based assay is the use of a microarray scanner for fluorescence readout that also improves on conventional well plate-based spectroscopic methods (45, 46). Because of these characteristics, the *nBio*Chip is ideally suited to antimicrobial susceptibility testing and ultra-high-throughput screening applications, including those designed to prevent biofilm formation and those designed to analyze established biofilms. It should be mentioned that, although we were able to estimate only the total microbial burden in our assays because of the limitations of the 2-wavelength scanner used in the study, the framework does facilitate distinguishing multiple species (using instruments such as a scanner with multiple lasers) on the basis of individual fluorescence.

From an evolutionary standpoint, the *nBio*Chip marks the beginning of the availability of the “ultra”-high-throughput biofilm assay with high-quality results that should be comparable to those obtained currently in standard industrial operations. Although a major obvious focus is high-throughput screening for antibiotic drug development, the platform has the potential to be expanded in the future to other high-content assays such as (i) screening of mutant and metagenomic libraries; (ii) high-content screening using peptides, aptamers, and nanoparticles; (iii) studies on the microbiome; and (iv) studies on biofilm-biomaterial interactions, where the microorganism can be encapsulated in a hydrogel of interest to mimic pathophysiological conditions. These assays can pave the way for the discovery of the next class of wonder drug, prophylactic agents, and adhesion-preventing molecules that can eventually serve as coatings on biomaterials such as catheters, stents, and other implants. The *nBio*Chip will serve as a high-throughput alternative to screening for analysis of highly potent combinations of all commercially available, FDA-approved drugs against any mixed-microbial-species biofilms.

## MATERIALS AND METHODS

### Culture conditions.

Frozen stocks of *Staphylococcus aureus* strain UAMS 1 and *Pseudomonas aeruginosa* strain PAO1 were subcultured onto tryptic soy agar (TSA) plates (BD Difco, MD) and were used to propagate the microorganisms in 10 ml of tryptic soy broth (TSB) (BD Difco, MD) in an orbital shaker at 37°C. In order to capture cells in log phase, a 100-μl volume of the overnight liquid culture was subcultured into 10 ml of TSB for 3 h. *Candida albicans* strain SC5314, stored at −80°C in glycerol stock, was cultured on yeast extract-peptone-dextrose (YPD) agar plates (BD Difco, MD) and incubated at 37°C for 24 h. A loopful of cells from the YPD plates was inoculated into 20 ml of YPD liquid media and grown in an orbital shaker at 30°C for 12 to 18 h; under those conditions, *C. albicans* grows as yeast cells.

### Microarray printing of nanobiofilms.

For a monobacterium biofilm chip, cells (grown as described above) were harvested, washed twice, and resuspended in sterile phosphate-buffered saline (PBS) (Sigma, MO) free of calcium and magnesium. Bacterial cells were adjusted to a density of 1 × 10^7^ cells/ml in a suspension of 1.5% alginate, 3× brain heart infusion (BHI) medium, and 2× yeast extract-peptone-dextrose (YPD) (BD Difco, MD) supplemented with 10% human serum (Invitrogen, WI). Using a noncontact microarray spotter (Omnigrid Micro; Digilab Inc., Holliston, MA), the aforementioned mixture was printed on functionalized polystyrene-co-maleic anhydride (PSMA)-coated glass slides at 30 nl per spot (12). An array of 48 rows and 12 columns was printed at room temperature and 100% relative humidity. In the case of the *S. aureus*-*P. aeruginosa* polymicrobial biofilm chip, the cell suspensions of the two species were adjusted to an equivalent density of 1 × 10^7^ cells/ml. For the *S. aureus*-*C. albicans* polymicrobial nanobiofilm chip, the bacteria and fungi were each adjusted to 5 × 10^6^ cells/ml and combined in a suspension consisting of 1.5% alginate, 3.3× RPMI 1640 (Corning; Cellgro, VA), and 1.2× YPD supplemented with 10% human serum. After printing was performed, the slides were placed in a humidified hybridization cassette (Arrayit, Sunnyvale, CA, USA) to prevent evaporation of spots and were incubated at 37°C. All microarrayer functions such as sample loading, priming, printing, and spatial distribution of the array were controlled by AxSys programing (Digilab, MA).

### Microscopy.

The morphology, spatial distribution, and confluence of the microcolonies and the architecture of the nanobiofilms grown were routinely monitored by confocal laser scanning microscopy. All fluorescent dyes used in this study were purchased from Life Technologies, Inc., Carlsbad, CA, USA. Using a combination of dyes, the bacterial population and the fungal population in each spot were distinguished and enumerated. Fluorescent dyes such as SYTO-9 and Live/Dead yeast were used to monitor the viability of the biofilms as a function of membrane integrity and metabolic activity. SYPRO Ruby Film Tracer was used to stain the exopolymeric matrix production of nanobiofilms. Wheat germ agglutinin (WGA)-Texas red was used to differentially stain the Gram-positive microbial population, and SYTOX green, a nucleic acid stain, was used to distinguish dead cells from the live cells. To determine the three-dimensional morphology of the nanobiofilms, SYTO-9 and SYPRO Ruby (for mono- and polybacterial biofilms) and FUN-1 and concanavalin A (for bacterium-fungus polymicrobial biofilms) were used and images were produced using an LSM 510 confocal scanning laser microscope (CSLM) (Carl Zeiss, Inc.).

### Characterization of mono-and mixed-species nanobiofilms.

Confocal laser scanning images of monospecies and mixed-species nanobiofilms were analyzed using IMARIS V 7.6 (Oxford Instruments, Zurich, CH) for segmentation and interpretation of microcolonies. Briefly, the spatial and population heterogeneity within a biofilm was characterized using the surpass mode by segmenting the image according to the color and intensity. The resulting segments were analyzed for geometric measurements and statistical relevance on the basis of the fluorescence intensity. The categories analyzed for this article were biofilm volume, shape, and compactness, microcolony count and volume, and exopolymeric matrix distribution. The measurements for the biofilm and for the exopolymeric matrix were performed on the basis of the use of SYTO-9 and SYPRO Ruby, respectively. The statistics determined on the basis of all values within a selected category codified the relative differences between categories observed in monospecies and mixed-species nanobiofilms.

### Viability assay.

The viability of nanobiofilms on the microarray was determined by staining with FUN-1 fluorescent dye. Although originally developed for the visualization of metabolically active fungal cells (which are able to process the dye), FUN-1 also binds to the plasma membrane of viable and nonviable bacterial cells (24). The excitation and emission spectra of FUN-1, 480 to 535/550 nm, are compatible with the filters installed in most microarray scanners. Briefly, the nanobiofilms were stained with 1 μM FUN-1 by immersion of the entire microarray slide in a staining jar and incubation in the dark at 37°C for 30 min. Following incubation, the *nBio*Chip microarray was washed in phosphate-buffered saline (PBS) by a simple dunk-and-rinse procedure to remove excess stain. After air-drying of the chip, a microarray scanner (GenePix Personal 4100A; Axon Instruments, Union City, CA) was used to scan for images. The microarray scanner images were analyzed with GenePix Pro V7 (Axon Instruments, Union City, CA) to determine the antimicrobial susceptibility profile, as described in references 12 and 21. Briefly, the percentage of viability of cells in response to different doses of different drugs was calculated from the fluorescence intensities of individual spots after setting the fluorescence intensities of live and dead controls at 100% and 0%, respectively.

### Antimicrobial susceptibility testing using nanobiofilm microarray.

Antimicrobial stock solutions of vancomycin hydrochloride (Sigma, MO), clindamycin hydrochloride (RPI Corp., IL), ciprofloxacin hydrochloride (Sigma-Aldrich, WY), tobramycin sulfate (Sigma, MO), methicillin sodium salt (Sigma, MO), and linezolid (AK Scientific, CA) were diluted in phosphate-buffered saline to a maximum concentration of 100 μg/ml. The stock solutions of the antifungal drugs amphotericin B and fluconazole were diluted in PBS to maximum concentrations of 16 μg/ml and 2 mg/ml, respectively. Any subsequent dilutions needed for the antimicrobial susceptibility assays were made in PBS. To study the effect of drugs in preventing biofilm formation, 50 nl of drugs was printed at 2-fold dilutions on top of the hydrogel spots after initial printing of the cells. The microarray(s) containing drugs was then incubated in a humidified hybridization cassette for a maximum of 24 h, gently washed in PBS, stained with FUN-1, and analyzed using the microarray scanner.

### Combinatorial screening of amphotericin B and fluconazole with vancomycin.

A freshly prepared stock solution of vancomycin was diluted to a final concentration of 100 μg/ml just prior to use, and 50 nl was spotted on top of the 50-nl cell spot printed on the microarray. Another iteration of printing was carried out to deposit 50 nl of amphotericin B (Sigma, MO) and fluconazole (Hospira, IL) at dilutions of 50, 5, 0.5, 0.05, and 0.005 μg/ml. The microarray was incubated for 16 to 18 h, stained with FUN-1, and analyzed for viability.
